# Therapeutic Obstinacy in End-of-Life Care—A Perspective of Healthcare Professionals from Romania

**DOI:** 10.3390/healthcare12161593

**Published:** 2024-08-10

**Authors:** Gema Bacoanu, Vladimir Poroch, Maria-Gabriela Aniței, Mihaela Poroch, Eliza Maria Froicu, Alina Mihaela Pascu, Beatrice Gabriela Ioan

**Affiliations:** 1 2nd Internal Medicine Department, Faculty of Medicine, Grigore T. Popa University of Medicine and Pharmacy Iasi, 700115 Iasi, Romania; gema.bacoanu@umfiasi.ro (G.B.); vladimir.poroch@umfiasi.ro (V.P.); eliza-maria.froicu@umfiasi.ro (E.M.F.); 2 Department of Palliative Care, Regional Institute of Oncology, 700483 Iasi, Romania; 3 Department of Surgery, Faculty of Medicine, Grigore T. Popa University of Medicine and Pharmacy Iasi, 700115 Iasi, Romania; 4 Department of Preventive Medicine and Interdisciplinarity, Faculty of Medicine, Grigore T. Popa University of Medicine and Pharmacy Iasi, 700115 Iasi, Romania; boanca.mihaela@umfiasi.ro; 5 Medical Oncology Department, Regional Institute of Oncology, 700483 Iasi, Romania; 6 Faculty of Medicine, Transylvania University of Brasov, 500036 Brasov, Romania; 7 Legal Medicine Department, Faculty of Medicine, Grigore T. Popa University of Medicine and Pharmacy of Iasi, 700115 Iasi, Romania; beatrice.ioan@umfiasi.ro; 8 Institute of Legal Medicine of Iasi, 700455 Iasi, Romania

**Keywords:** therapeutic obstinacy, futile treatment, end-of-life care, health-care professionals, palliative care

## Abstract

Background: End-of-life care raises ethical, moral, legal and economic dilemmas, especially when physicians have to decide whether to initiate or to stop treatments that may be considered disproportionate and futile. Aim: To explore the opinion of health care professionals involved in end-of-life patient care on interventions considered disproportionate and futile at this stage of care, the causes and factors of pressure leading to such situations, and possible solutions to reduce the phenomenon. Material and method: The study used an adapted, designed questionnaire intended for health professionals caring for patients at the end of life. The 128 respondents were physicians, nurses, psychologists and social workers who expressed their opinions about therapeutic obstinacy in end-of-life care. Results: The results of the research highlight the role of the family as a pressure factor, the causes related to the non-acceptance of the prognosis and diagnosis of a terminal condition, fear of death and ignorance of the patient’s wishes. Interventions considered disproportionate at the end of life were cardiopulmonary resuscitation, mechanical ventilation, transfusion of blood derivatives, complex diagnostic investigations and the establishment of gastrostomy/jejunostomy in the last days of life. Conclusions: End-of-life therapeutic obstinacy is a reality in end-of-life care, and healthcare professionals face many ethical challenges in this process. Care decisions must be made together with the patient and their family, respecting the rights, dignity and respect of all parties involved in the process.

## 1. Introduction

End-of-life patient care is becoming an increasingly important topic in today’s medicine. Although death is a fact of our lives, medicine has made important technological progress in sustaining and prolonging life expectancy [1]. In this context, therapeutic obstinacy and futile treatments at the end of life represent a reality in medical practice and are topics of debate with ethical, moral, professional, legal and economic implications [2].

Therapeutic obstinacy is “the initiation or continuation of medical actions that have no other purpose than prolonging the life of a patient facing irreversible death” [3]. Therapeutic obstinacy induces the illusion of a prolonged life for patients who have no chance of recovery or minimal preservation of life quality, and the treatment applied becomes useless and burdensome [4].

**Figure 1:**
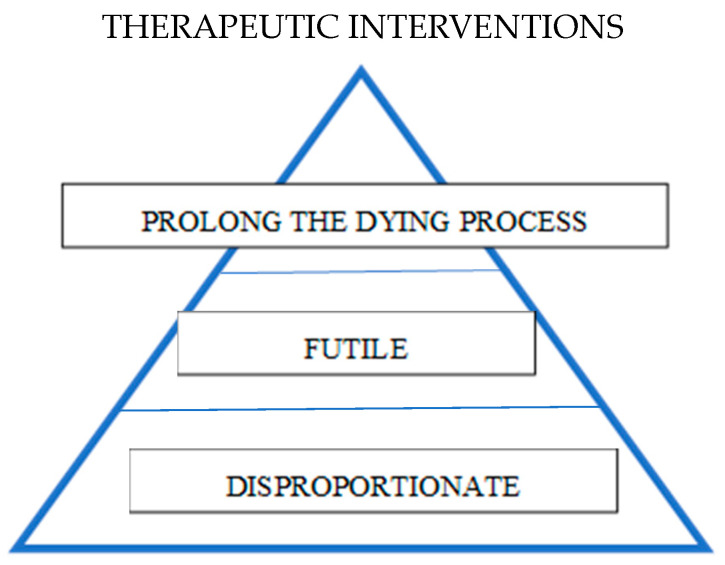
Pyramidal representation of end-of-life intervention assessment criteria in the context of therapeutic obstinacy (adapted according to Barragán JL. [5]).

According to Barragan [5], interventions/therapies at the end of life may be considered therapeutic obstinacy when they meet the following conditions: they are disproportionate in relation to the risk-benefit balance, they are futile, and they actually prolong the dying process. They do not provide a cure, nor do they improve the quality of life of the patient or his/her family. Disproportionality is judged individually by the physician, the patient, the patient’s family, society, and bioethics committees, by all these actors together if possible. The pyramidal arrangement of criteria for evaluating these interventions highlights their hierarchy in the evaluation process, according to Barragán JL. [5]. (see Figure 1) This figure shows the importance of the disproportionality from the base of the pyramid to the top as a criterion in evaluating the appropriateness of certain interventions for these patients at the end of life.

For palliative care professionals, deciding when to discontinue or not to initiate futile treatments could represent a difficult dilemma. Conflicts can arise when physicians and patients or their families have different perspectives on the goals of care, especially when patients or their families request futile therapies [6]. Moreover, health care professionals’ (HCPs’) opinions may differ on the causes and the factors of pressure that can lead to therapeutic obstinacy at the end of life and on the medical interventions that may be included in this category [7].

Among the identified causes that can lead to such situations are related to physician’s orientation towards curative treatment, their lack of experience with death and the dying process, concerns about legal, ethical and professional risks, their poor communication skills, personal factors related to adherence to various religious beliefs, fear and worries, structural and functional deficiencies of ethics committees, as well as insufficient palliative care services and facilities. Other causes also relate to patient and family requests for further treatment, uncertainty of prognosis, lack of information about patient wishes, and patient and family fears of loneliness and neglect [7,8]. The opinions of HCPs on these issues may vary due to their different training, roles and levels of responsibility towards health institutions and the care process [4,7,9].

According to the published data, the most common end-of-life interventions subject to ethical debate are the opportunity to initiate/continue artificial nutrition and hydration, mechanical ventilation, antibiotic therapy, chemotherapy, any surgery, dialysis, invasive diagnostic procedures, cardiopulmonary resuscitation, mechanical circulatory support [9,10,11,12]. 

A systematic review published in 2016 [4] shows that futile treatments in the Intensive Care Unit (ICU) may have a negative impact on job satisfaction and can induce emotional exhaustion in physicians and nurses, with negative consequences for patients and their families [4].

In Romania, end-of-life care has specific social, cultural, legal and economic peculiarities. Thus, the long-term care of the elderly and people at the end of life is mainly carried out in an informal system, with the involvement of family members, neighbours and the patient’s community of which the patient is a part. Also, the end-of-life care process has integrated a deep sense of the Orthodox-Christian tradition as a way of promoting dignity at the end of life and death itself. The continued development of specialized palliative care services will ensure, over time, the transfer of the patient to various formal care systems. The legal framework of care at the end of life is represented by the legislation regulating the organization, operation, and authorization of palliative care services in Romania, to which medical protocols specific to palliative care are added per international protocols. However, there are many vulnerabilities and ethical challenges in end-of-life care, highlighting the need for regulations that favour respect for patient dignity and autonomy in end-of-life treatment decisions [13,14].

The aim of this research is to explore the opinion of HCPs involved in end-of-life patient care on interventions considered disproportionate and futile at this stage of care, the causes and factors of pressure leading to such situations, and possible solutions to reduce the phenomenon. 

The results of our research are of interest to palliative care, oncology and critical care practitioners in ICU and emergency medicine, as well as to those intending to practice in these areas, and will provide contributions to debates on ethical issues in end-of-life care. In Romania, these topics have not been explored so far, and the poor legislative framework in this area makes it difficult for HCPs to manage situations that can be characterized as therapeutic obstinacy at the end of life.

## 2. Materials and Methods

To achieve the proposed aim, we conducted a quantitative study in which data collection was carried out using a questionnaire specially designed for this research (see Annex S1 from the Supplementary Materials). As a result of the fact that palliative care in Romania is still insufficiently developed in relation to the needs of the population and unevenly distributed in the territory, the “snowball” method was used in the selection of the target group, which allowed the recruitment of study participants. This method is suitable for small groups that are difficult to identify [15]. Twenty-seven specialized medical and socio-medical units from different regions of Romania were identified, and a letter of intent was sent to the institution manager. The invitation explained the purpose and objectives of the research, the people who can participate in the study, the way to complete the questionnaire (in writing or online- via the specified link) and the way to submit the documents.

The questionnaire was self-administered and was made available to study participants in both hard copy and online format [16]. Participants were informed of the choice between a paper and an online questionnaire at the time of recruitment and in the information materials. No conflicts of interest were identified between researchers or participants. Paper questionnaires were collected in sealed envelopes and stored in secure locations until data entry into the electronic system. Only authorized researchers had access to these questionnaires. Participants were allowed to skip any question they did not wish to answer without consequence. Participants had the opportunity to withdraw from the study at any time without any negative consequences.

The data were collected between June and November 2023.

The study group was selected from HCPs caring for patients on end-of-life or members of palliative care teams: physicians, nurses, psychologists and social workers. 

The inclusion criteria were an agreement to participate in the research and meeting one of the following conditions: professional experience in caring for patients on end-of-life or membership in the palliative care team. The participants self-reported their professional experience and verified it by declaring their place of work.

The exclusion criteria were HCPs who did not have experience with the care of patients at the end of life or those who did not agree with the processing of personal data. 

### 2.1. Validity and Reliability of the Questionnaire

The design of the questionnaire started from the identification of relevant items for this phenomenon in the literature [4,6,7,8]. Subsequently, the items were refined on the basis of the results of three focus groups and two pretests (pilot surveys) so that the questionnaire would provide valid, accurate and reliable data. In this way, the questionnaire created is adapted to the social, cultural and medical conditions in Romania [17].

The questionnaire is structured in three parts. The first part of the questionnaire collects the sociodemographic and professional data of the study participants (5 questions). The second and third parts of the questionnaire comprise 20 questions, of which 19 questions are close-ended, multiple-choice questions, and only the last question is open-ended, giving participants the opportunity to express their opinion on the topic under investigation. The second part of the questionnaire (Q1–Q12) explores the knowledge of the participants about the concept of therapeutic obstinacy at the end of life, assesses the frequency of encountering such situations in everyday practice (Q1–Q3), assesses the pressure factors on the medical staff and the causes of this phenomenon (Q5–Q7), evaluates the possible solutions to reduce this phenomenon (Q8, Q10–Q12), as well as which are the most frequent interventions that can be evaluated as disproportionate at the end of life (Q4, Q9). The third part of the questionnaire explores the opinion of the study participants regarding the importance of psychological and spiritual support for the patient, his family and the medical staff in the process of accepting the patient’s death (Q13–Q18) as well as what are the most frequent wishes of the patients at the end of life (Q19) (See Annex S1 from the Supplementary Materials).

The Cronbach’s Alpha values for questions 5 (Q5), question 6 (Q6), question 9 (Q9), question 10 (Q10), question 11 (Q11) and question 19 (Q19), are well above the generally accepted threshold of 0.7, indicating that the items within each construct are highly reliable [18]. There were six items related to the subquestion that examined specific constructs; Cronbach’s alpha was used to determine the reliability of these items to measure the examined construct. This suggests that the items (subquestion) used for this questionnaire are consistent and dependable for measuring the intended questions (see Table 1).

The collected data were centralised and analysed using SPSS 18.0 [19]. The analysis tool used to decode all the information contained in the collected data was Correspondence Factor Analysis (CFA). By using this method, one can see, observe and understand how different categories of responses are interrelated, thus developing a better understanding of the dynamics of inter-variable interactions [19]. 

In the first stage, a correspondence matrix or contingency table was developed, reflecting how often different combinations of categories occur in the variables studied. The next step in the CFA was to calculate its own values and vectors in relation to the correspondence matrix. Using only a few principal dimensions, the method simplified the datasets while retaining relevant information about them.

### 2.2. Graphical Representation of the Data

Dimensions are created based on the values of each response variable frequency-never, rarely, rare, often, very often. Thus, dimension 1 corresponds to the lowest values of each variable frequency, while dimension 2 corresponds to the highest value of each response variable frequency. Once the principal dimensions have been derived, a geometric space has been formed in which categories of variables and respondents are represented as points. Such a visualisation helps to discover the links between categories and how they can be seen in a multidimensional environment. The closeness of two groups of data in a chart indicates their similarity or relationship, and increasing distances signify differences or separation. Studying these relationships, on the other hand, provides valuable information about how the data are organised, making it possible to identify associations or discriminations between different groups. 

The significance of the presence of associations between groups was analysed using the Chi-square test by comparing probability (sig) with a 10% significance threshold. The sizes for each correspondence analysis were chosen by choosing the smallest number of categories of a variable minus one. The significance threshold for Chi-square analysis was set at *p* < 0.10. For Correspondence Factor Analysis (CFA), we also used a significance threshold of *p* < 0.10 to determine the significance of associations between variables.

## 3. Results

The questionnaire was completed by 137 professionals. Of the questionnaires received, nine questionnaires were discarded because they were not fully completed. Therefore, 128 questionnaires were considered in the data analysis.

### 3.1. Participants

Participants in the study were 128 HCPs caring for patients on end-of-life. Of these, 51 were physicians of various specialties (11 oncologists, 8 family physicians, 4 psychiatrists, 4 internal medicine, 2 pneumologists, 2 diabetologists, 2 emergency medicine, 1 ICU, 2 oncological surgeons, 2 neurologists, 1 haematologist, 1 occupational medicine, 1 infectious disease, 1 radiotherapy, 9 unclassified specialties), 65 nurses, 7 social workers and 5 psychologists. Women constituted 78.9% of the participants. Professional experience (expressed in years) in caring for patients at the end of life was balanced across the four categories (Table 2).

The results of the opinion of HCPs are presented on the knowledge of the concept of therapeutic obstinacy at the end of life, the frequency of encountering such situations in practice, the personal experiences (with relatives/friends) that can be classified as therapeutic obstinacy at the end of life, the factors of pressure from the patient’s family and the causes of this phenomenon. The opinions of HCPs are presented on what end-of-life interventions may be considered disproportionate and futile in relation to the risk-benefit balance and what the possible solutions are to fight/reduce this phenomenon. All these results are correlated with the professions of the study participants: physicians, nurses, psychologists, and social workers.

### 3.2. Knowledge of the Concept of Therapeutic Obstinacy in the Study Group

To the question, “*Are you familiar with the concept of end-of-life therapeutic obstinacy?*” (Q1), the majority of respondents, 119 responders (93% of the total valid responders), indicated that they were familiar with the concept of therapeutic obstinacy and only 9 respondents (7%), were not familiar with this concept (Table 3).

### 3.3. Frequency of End-of-Life Therapeutic Obstinacy Encountered in Daily Practice 

When asked about “*How often do you think you encounter situations of therapeutic obstinacy in practice?*” (Q2), 77 participants (60.2%) responded that they often encounter these situations in practice. Physicians and nurses most often encounter therapeutic obstinacy situations. Psychologists and social workers rarely encounter these situations (Table 4).

When asked, “*Have you had personal experiences (with relatives/friends) that can be categorised as “end-of-life therapeutic obstinacy?*”, 30.7% of the respondents said they rarely had such experiences, 15.7% never, 29.9% rarely, 22% often, and 15.7% very often.

Of the 28 subjects (22%) who said they often had personal experiences that could be categorised as “therapeutic obstinacy at the end end-of-life of life”, 71.4% were female, 39.3% were aged 41–50, 60.7% were nurses, 57.2% had less than 10 years’ professional experience.

### 3.4. Factors of Pressure from the Patient’s Family and Causes Leading to Therapeutic Obstinacy at the End of Life

The factors of pressure from the patient’s family that can lead to therapeutic obstinacy at the end of life on which the study participants expressed their opinion were with regard to the family’s background (urban/rural/abroad-diaspora), the patient’s gender (female/male), the patient’s age (<18 years/18–45 years/45–65 years/65–75 years/>75 years), the family’s level of education (no education/elementary education, secondary education, higher education), the family’s degree of religiosity (non-religious/religious), as well as the nature of the patient’s condition (oncological/non-oncological), the patient’s ethnicity, and the wish to see the loved one for the last time.

To the question, “*What do you consider to be the factors of pressure from the patient’s family in situations of therapeutic obstinacy?*” (Q5), correspondence analysis demonstrates a significant relationship between medical occupation (D2) and the perception that family pressure influences therapeutic decisions, demonstrated by the statistical significance shown in Annex S2 from the Supplementary Materials.

Regarding the patient’s family origin (rural, urban, or abroad/diaspora), physicians and nurses frequently deal with pressure from the patient’s family in situations of therapeutic obstinacy, while psychologists and social workers encounter this pressure less frequently. The frequencies are categorized as Never, Very Rare, Rare, Often, and Very Often. 36% of physicians stated that they often encounter pressure from family members abroad/diaspora; 28% report encountering this pressure rarely, 28% very often and 8% encounter it very rarely. Nurses reported that 33.8% often encounter pressure from family members in the abroad/diaspora, 18.5% very often, 27.7% rarely and 16.9% encounter it very rarely. Physicians appear to be the ones who most frequently perceive pressure from diaspora families in medical practice, whereas psychologists and social workers feel it the least. Nurses fall somewhere between the two extremes, with a variety of perceptions about the frequency of this type of pressure.

Regarding the patient’s age as a pressure factor, the results show that the young age of the patient (under 18 years and the age category 18–45 years) is perceived by the medical staff as a pressure factor. Physicians stated that 34% rarely encounter pressure from patients under 18 years, 30% very often, 28% often, 6% never and 2% very rarely encounter this pressure. Nurses stated that 41.5% often encounter this pressure, 2.3% rarely, 15.4% very often and 10.8% very rarely. Psychologists and Social Workers stated that 41.7% very often encounter pressure from patients under 18 years old, 25% rarely, 25% never encounter this pressure, and 8.3% very rarely encounter it. Physicians often feel pressure from families of young patients and children. Psychologists and social workers rarely feel such pressures, regardless of the patient’s age. Nurses oscillate between feeling such pressures more often than psychologists but less often than physicians. 

Religiosity of the patient’s family as a factor of pressure: There are variations in how health professionals perceive the pressure exerted by non-religious families. Physicians see it often and very often 36%, psychologists and social workers rarely and very rarely 1.6% and never 33.3%. Nurses have divided opinions, ranging from rarely 43.1% to very often 6.2%. 

With regard to patient ethnicity, the study participants specified that it was often (18.8%) and very often (16.4%) a pressure factor for therapeutic obstinacy at the end of life, the most common ethnicity specified being of Roma ethnicity. 

### 3.5. Causes of Therapeutic Obstinacy Phenomenon (Q10)

The causes of therapeutic obstinacy phenomenon at the end of life on which the study participants expressed their opinion were: (a) lack of knowledge of the diagnosis of the basic disease by the patient, family and physician/health care professional, (b) lack of knowledge of the diagnosis of the terminal condition by the patient, family and physician/health care professional, (c) non-acceptance of the diagnosis of the terminal condition by the patient, family and physician/health care professional (d) lack of knowledge of prognosis of illness by patient, family and physician/health care professional, (e) non-acceptance of prognosis by patient and family, (f) short time after diagnosis, (g) emotional storm in the context of diagnosis or prognosis, (h) poor collaboration/communication between physician and patient/family, (i) poor collaboration/communication between nurse and patient/family, (j) degree of religiosity of patient/family/physician, (k) fear of death of patient/family/physician, (l) fear of death of patient/family/physician, (m) non-expression of patient’s end-of-life preferences, (n) lack of knowledge of patient’s end-of-life preferences by family/physician, (o) fear of HCPs of malpractice claimsed by patient/family/patient. 

Correspondence analysis demonstrates a significant relationship between medical occupation and the perception of the causes of the occurrence of therapeutic obstinacy phenomenon; this is demonstrated by the statistical significance shown in Annex S2 from the Supplementary Materials. 

Regarding the causes that lead to therapeutic obstinacy, health professionals pointed out that lack of knowledge of the diagnosis and the diagnosis of the terminally ill by the patient and his family may underlie the demand for disproportionate interventions. 42% of physicians often encounter therapeutic obstinacy due to the patient’s lack of knowledge about their prognosis, 26% rarely encounter this issue, 22% very often, and 10% very rarely. 38.5% of nurses deal with therapeutic obstinacy often due to the patient’s lack of knowledge about their prognosis, 21.5% very rarely, 18.5% rarely and 21.5% very often. 

Regarding the emotional impact of the diagnosis, physicians rarely consider that it leads to obstinacy, nurses encounter such cases variably, and psychologists and social workers encounter it occasionally.

**Figure 2:**
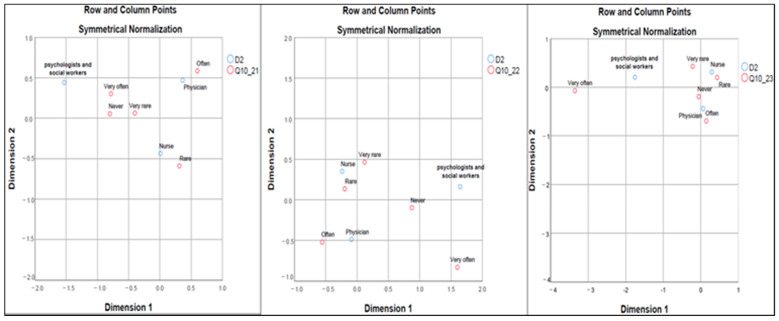
Correspondence maps between professions (D2) and patient and family religiosity (Q10_21, Q10_22) and physicians’ religiosity (Q10_23); Dimension 1—corresponds to the lowest values of each variable frequency (never, rarely, rare, often, very often); Dimension 2—corresponds to the highest value of each response variable frequency (never, rarely, rare, often, very often); Q10_21—a low degree of patient’s religiosity; Q10_22—a low degree of family’s religiosity; Q10_23—a low degree of the physician’s religiosity; D2—profession.

Figure 2 illustrate that HCPs express the impact of religiosity (Q10_21) on therapeutic obstinacy differently. The physicians are more likely to report religiousness-related obstinacy in patients and their families (Q10_22), placing them closer to the “Often” frequency on the maps. Nurses perceive this impact as less frequent, falling between “Very rarely” and “Rarely”. In contrast, the personal religiosity of the physician (Q10_23) is not seen as influencing care decisions, with all professions clustering around the “Never” point.

**Figure 3:**
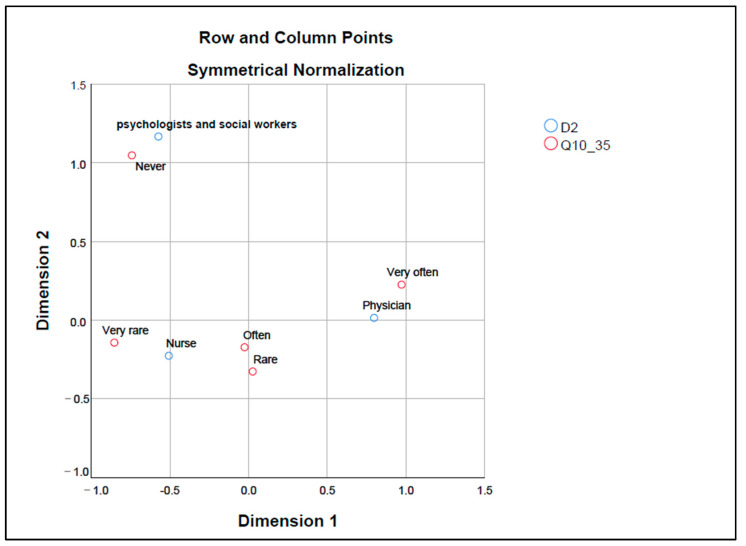
Fears of malpractice complaints by medical staff from patient’s family; Dimension 1—corresponds to the lowest values of each variable frequency (never, rarely, rare, often, very often); Dimension 2—corresponds to the highest value of each response variable frequency (never, rarely, rare, often, very often); Q10_35—fear of medical staff regarding malpractice complaints from patient’s family a low degree of patient’s religiosity; D2—profession.

Correspondence map in Figure 3 shows the relationships between professions (D2) and the frequency of perceived fears of medical staff of malpractice complaints from the patient’s family as a cause of therapeutic obstinacy (Q10_35).

Psychologists and social workers and the “Never” category are placed close to each other, indicating that they tend not to perceive fear of malpractice claims as a cause of therapeutic obstinacy. Nurses are close to “Very rarely” and “Often”, suggesting a variation in the perceived frequency of fear of malpractice complaints as a cause of therapeutic obstinacy. Physicians are close to the “Very often” category, indicating a high frequency of these fears among physicians. The “Rarely” category is positioned between nurses and physicians, suggesting that it is perceived as a cause of therapeutic obstinacy occasionally, but not as intensely as “Very often”.

The map suggests clear differences between professions in how fear of malpractice claims is perceived as a factor in therapeutic obstinacy. Physicians appear to be most concerned about it, psychologists and social workers are the least concerned and nurses fall between the two extremes with a more varied perception.

### 3.6. End-of-Life Interventions That May Be Considered Disproportionate to the Benefits Achieved and the Patients’ Quality of Life 

For the question, “*In caring for the patient in the last 72 h of life, which interventions do you consider to be disproportionate to the benefits obtained?*”, Table 5 shows respondents’ perceptions of the frequency with which certain medical interventions are considered to be disproportionate to the benefits achieved for patients on the end-of-life. Responses are distributed on a scale from “Never” (1) to “Very often” (5).

Cardiopulmonary resuscitation is seen as often or very often disproportionate (accounting for 66.4% of responses), indicating a strong perception that the intervention may be too aggressive relating to the benefits it brings to patients in the last 72 h of life.

Regarding mechanical ventilation, 56.2% of respondents believe it is often or very often disproportionate, reflecting the concern that the benefits may be limited in relation to the invasiveness and associated discomfort.

Blood derivative transfusion has a total of 49.2% of responses in the “Often” and “Very often” categories, and there is a concern that this treatment may be more harmful than beneficial in the last days of life.

For parenteral nutrition, 45.7% of respondents “Often” or “Very often” consider it disproportionate, suggesting scepticism about the benefits of intravenous nutrition in the terminal setting.

Parenteral hydration is seen as similar to parenteral nutrition, with a slight tendency to be more commonly seen as disproportionate.

For antibiotic therapy, the distribution of responses suggests that there is a split in the perception of antibiotic therapy use; some professionals see a risk of overuse in the context of limited benefit.

For haemodialysis, a fairly even distribution of responses is observed, but with a greater tendency to be frequently disproportionate.

Complex diagnostic investigations (CT/MRI/PET-CT), with 51% of responses in the “Often” and “Very often” range, reflect an awareness that such investigations may be too burdensome for patients without a clear end-of-life benefit.

For paracentesis and thoracentesis, responses indicate that these procedures are considered among the most disproportionate, with almost 55% of respondents indicating “Often” or “Very often”.

Overall, responses indicate a perception that many intensive or invasive interventions are frequently disproportionate in the context of palliative care at the end of life. Also, according to the analysis of the obtained data, there was no significant association between professions and the application of disproportionate treatment in the last 72 h of life. 

To the question “*What do you think would be the solutions to avoid situations of therapeutic obstinacy?*” (Q11), the most frequent solution mentioned by respondents was better communication between members of the medical team and the patient or his/her family (51.6%) and, also, with the patient (48.4%). Respondents also mentioned that possible solutions include better communication between the patient and the nurse (43%), patient and psychologist (45.3%), and priest/spiritual assistant (42.2%). 

Informed consent explained and assumed by the patient and the patient’s family, at the patient’s wish, was appreciated by 71.9% and 74.3% of respondents, respectively, as “Often” and “Very often” the solution to avoid situations of therapeutic obstinacy at the end of life.

70.3% of respondents believe that legislative regulation is “Often” and “Very often” needed regarding when cardiopulmonary resuscitation (CPR) is not recommended at the end of life, and 72.7% of respondents believe that medical procedures and guidelines are “Often” and “Very often” needed for these situations. Also, 79.7% think that training is “Often” and “Very often” important in avoiding these situations (see Table 6).

**Figure 4:**
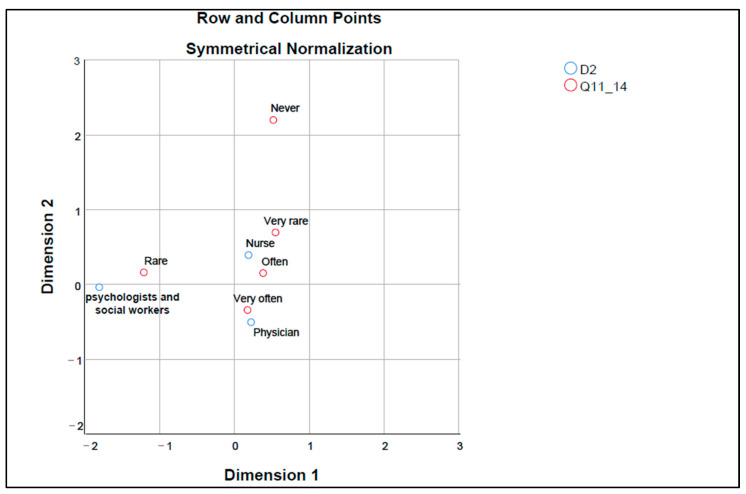
Perception of the usefulness of medical procedures and guidelines as a solution to avoid therapeutic obstinacy solutions; Dimension 1—corresponds to the lowest values of each variable frequency (never, rarely, rare, often, very often); Dimension 2—corresponds to the highest value of each response variable frequency (never, rarely, rare, often, very often); Q11_14—Medical procedures and guidelines for these situations; D2—profession.

The correspondence map in Figure 4 explores the relationship between the different healthcare professions (D2) and their perception of the usefulness of medical procedures and guidelines as a solution to avoid situations of therapeutic obstinacy (Q11_14).

Psychologists and social workers are close to “Rarely”, indicating that they perceive the usefulness of medical procedures and guidelines in situations of therapeutic obstinacy as less frequent. Physicians are close to “Very often”, suggesting that they regularly see medical procedures and guidelines as an effective solution in dealing with therapeutic obstinacy. Nurses are close to “Often”, indicating that they also see medical guidelines and procedures as usually helpful in these situations, but perhaps not as strongly as physicians. The” Never” and “Very Rarely” categories are isolated and located away from either profession, suggesting that most medical professionals recognize the usefulness of medical guidelines and procedures in managing therapeutic obstinacy.

**Figure 5:**
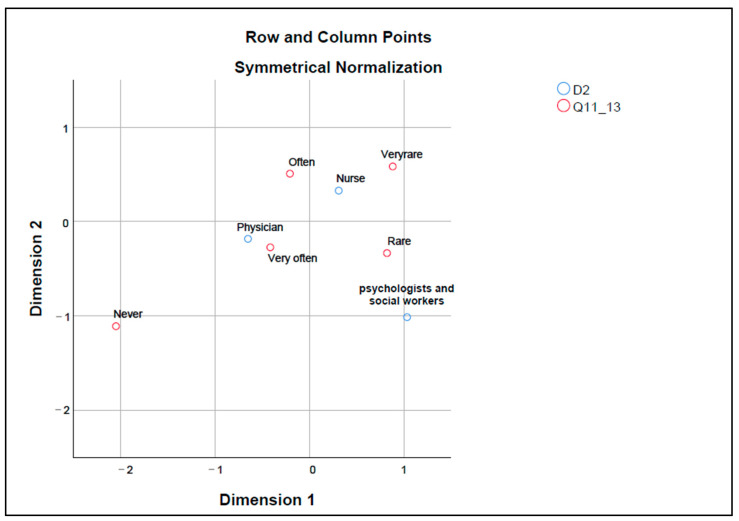
Map of correspondence between professions (D2) and perception of the solution “Legislative regulations on when CPR is not recommended” (Q11_13). Dimension 1—corresponds to the lowest values of each variable frequency (never, rarely, rare, often, very often); Dimension 2—corresponds to the highest value of each response variable frequency (never, rarely, rare, often, very often); Q11_13—Legislative regulation of when CPR is not recommended; CPR—cardiopulmonary resuscitation; D2—profession.

Legislative regulations on when cardiopulmonary resuscitation is not recommended are seen as a solution for managing situations of therapeutic obstinacy by both physicians and nurses participating in the study. Correspondence map number 5 (Figure 5) shows the relationship between the different health professions (D2) and the frequency with which it is encountered as a solution for managing therapeutic obstinacy (Q11_13).

Psychologists and social workers are placed close to “Rare”, suggesting that this professional category perceives this avoidance of therapeutic obstinacy as being necessary occasionally. Physicians are positioned centrally in the chart, close to “Very often”, which may indicate that they perceive this solution as frequently necessary in their practice. Nurses are close to “Often” and “Very rarely”, which may reflect a variation in nurses’ perceptions of the frequency of choosing the specified solution of therapeutic obstinacy.

To the question, “Do you agree that continuing treatment is disproportionate to the benefits in the last 72 h of life?” 17.5% of the subjects answered “Never”, 33.3%—“Very rarely”, 23.8% “Rarely”, 22.2% “Often”, and 3.2% “Very often”. 

Of the 42 subjects (33.3%) who very seldom agreed with continuing treatment disproportionate to the benefits in the last 72 h of life, 66.7% were female, 40.5% aged 41–50 years, 57.1% were nurses, 35.7% with 11–20 years of work experience.

## 4. Discussion

The aim of this research is to explore the opinions of HCPs involved in end-of-life care about interventions considered disproportionate and unnecessary at this stage of care, the causes and pressure factors leading to such situations, and possible solutions to reduce the phenomenon.

The professionals participating in the study responded that they are aware of the concept of therapeutic obstinacy at the end of life and that they frequently encounter this phenomenon in their daily practice. This is also in line with other studies that highlight that the HCPs are aware of therapeutic obstinacy and the problems it causes, that it is not uncommon in everyday practice for patients to be treated in this way, even if the therapy is potentially futile [8,9,20,21].

The pressure factors from the patient’s family that lead to therapeutic obstinacy at the end of life are multiple. As regards the environment of origin, urban and diaspora are pressure factors, which may be the consequence of the migration of the Romanian population in recent years (from rural to urban and abroad) [22]. The physical distance between patient and family can lead to partial knowledge of medical realities and the patient’s wishes and to requests for interventions disproportionate to the benefits for the patient’s quality of life and life expectancy and even to what the patient would have wished at this stage of his/her life. The patient’s wish expressed as a directive in advance helps in the end-of-life care decision-making process and expresses the patient’s autonomy in this process. The directive implies the designation by the patient of a person who can make decisions in his/her place when he/she is no longer able to do so [23]. In Romania, the lack of such regulations makes it difficult for the family and medical professionals to interact in such dilemmatic situations.

Previous studies point out that after learning of the patient’s terminal diagnosis, the patient’s family experiences a period of high stress that can manifest itself in anger, depression, interpersonal conflicts and psychosomatic problems and may feel hopelessness, anger, guilt and helplessness as they cannot alleviate the suffering of their terminally ill family member. Thus, from the perspective of the principle of autonomy, the patient, rather than the family, trustee or physician, makes the best decisions about limiting treatment or treatments that do not provide a cure but prolong life for a period of time [24,25].

Regarding the patient’s age as a family pressure factor, our results are consistent with previous studies that demonstrate that end-of-life medical decision-making is more ethically and legally complicated when dealing with children who are near death [26]. 

With regard to ethnic minorities, previous studies highlight influences on end-of-life care decisions related to religious beliefs, cultural issues leading to different understandings about changing treatment regimes, stopping aggressive therapy, the initiation of palliative care, the choice of a place for end-of-life care, the selection of preferred life-sustaining treatments such as artificial nutrition or mechanical ventilation, the desire for resuscitation and understanding of advance directives, and the understanding of living wills [27].

In our study, Roma ethnicity was the most common ethnicity specified as a pressure factor for therapeutic end-of-life care. Previous studies highlight the influence of strong family values and decision-making within these communities, the power of beliefs and superstitions related to illness, care and rituals related to death and dying. Difficulties in the care process also include problems of communication, awareness, limited access to services, and insufficient training of professionals to understand the cultural particularities of this ethnic group [28].

The gender of the patient, male or female, does not seem to influence family pressure on end-of-life care decisions, nor does family religiosity or the nature of the patient’s condition (oncological/non-oncological).

The degree of schooling of the patient’s family members is considered a pressure factor by the study participants and is directly proportional to the high level of schooling of the patient’s family. Higher education of relatives was rated by participants as a high level of pressure factor on the phenomenon of therapeutic obstinacy at the end of life. Thus, the high level of education of the patient’s family members requires staff training strategies to know how to communicate and explain these end-of-life care concepts [29].

Interventions considered to be disproportionate at the end of life valued by the professionals participating in the study were cardiopulmonary resuscitation, mechanical ventilation, transfusion of blood derivatives, complex diagnostic investigations (CT/MRI/PET-CT), and establishment of gastrostomy/jejunostomy for nutrition and hydration in the last 72 h of life. These types of interventions are in line with published data, which most frequently subjects to ethical debate the appropriateness of initiating or not initiating end-of-life artificial nutrition and hydration, ventilation, antibiotic therapy, chemotherapy, any surgery, dialysis, diagnostic and therapeutic interventions, cardiopulmonary resuscitation, mechanical ventilation, mechanical circulatory support, discontinuation and withdrawal of treatment [9]. 

As regards the solutions to reduce the phenomenon of therapeutic obstinacy at the end of life, one of the solutions mentioned by the respondents was better communication between physician–patient and family. Respondents also stressed the importance of holistic communication at the end of life, involving the nurse, psychologist and priest/spiritual assistant. In this respect, previous studies show that palliative medicine is an invaluable aid in the difficult goal of improving communication between physicians, patients and patients’ relatives during the course of the illness and especially at the end of life [23]. Psychoemotional and spiritual support at the end of life gives a sense of comfort and security to patients and their families and helps to defuse some religious and non-religious attitudes, with medical staff playing an important role in this supportive care [30]. 

The importance of informed consent is explained and assumed by the patient and the patient’s family, at the patient’s request, the development of legislative regulations on cases in which cardiopulmonary resuscitation is not recommended at the end of life and the implementation of medical procedures and guidelines for these situations were other solutions to avoid situations of therapeutic obstinacy at the end of life. Previous studies highlight that advance directives could help patients with advanced cancer through the end-of-life treatment decision process, as well as communicating preferences regarding DNR and other end-of-life interventions [25].

In Romania, healthcare professionals feel the lack of these legislative regulations on decision-making in end-of-life care. At the European level, attempts are being made to improve and standardize this process by proposing guidelines and legislative measures to be implemented by each EU Member State. In this regard, a guide intended to help healthcare professionals, the Guide for Decision-Making in End-of-Life Care, represents a source of information and a basis for discussion for patients, their families and all those involved with end-of-life care situations [31].

However, in clinical practice, it is very difficult to decide what is truly therapeutic obstinacy, requiring a high degree of professional competence, clinical experience, a sense of proportion, serenity, the continuous updating of therapeutic guidelines and the ongoing progress of clinical research in the field [32].

As end-of-life is a unique phase in the life course, previous studies have shown that perceptions of therapeutic obstinacy highlight the difficult and challenging situation of providing care at the end of life [7,8,9,10,11,12]. In our view, differing views can lead to varied discussions in a positive environment that considers different perspectives, experiences and beliefs and, at best, ends with a well-considered decision.

Study limitations: The present study does not capture the point of view of patients and their families on situations of therapeutic obstinacy, the reasons that might lead to such situations, and possible solutions they identify for optimising decision-making in end-of-life care. Another limitation of the study is the unequal collection of answers from different regions of the country, depending on the availability of the participants and the still unequal development of specialized palliative care services in Romania. The participants self-reported their professional experience and verified it by declaring the place of work, which may introduce a bias and represent a limitation of the study. The presence of partially completed questionnaires led to the exclusion of some respondents, which may represent a limitation of our study. 

## 5. Conclusions

Therapeutic obstinacy at the end of life is a reality in end-of-life patient care, and HCPs face many ethical challenges in the process. Care decisions must be made with the patient and their family, and the rights, dignity, and respect of all parties involved in the process must be respected. Healthcare professionals have no legal, ethical, moral or deontological obligation to provide futile therapies at the end of life.

There are multiple causes and factors contributing to pressure for therapeutic obstinacy at the end of life. The knowledge of these and the possible solutions identified by the participants in the study to fight/reduce this phenomenon can be a starting point for ethical, medical, economic and legal debates and legislative proposals to improve the cultural and medical context of end-of-life care in Romania, and for the development of educational resources for professionals in the field.

Interventions to reduce this phenomenon are based on better communication between members of the care team, the patient, and the patient’s family, as well as a holistic approach to the patient and the patient’s family. Adequate professional training of the medical team to optimally manage these situations should be a permanent concern of healthcare providers. 

Legislative changes to the regulation of end-of-life care decision-making, such as the communication of preferences regarding “do not resuscitate” (DNR) and other interventions, as well as the regulation of advance directives, can be a real help to healthcare professionals to avoid or manage these situations effectively, reducing the negative impact of end-of-life care burden on healthcare professionals, patients and their families, as well as on the Romanian healthcare system.

## Figures and Tables

**Table 1:** The Cronbach’s Alpha values for Constructs Related to Therapeutic Obstinacy.

Reliability Statistics		
Construct	Cronbach’s Alpha	Number of Items
Q5	0.867	19
Q6	0.917	17
Q9	0.932	15
Q10	0.940	35
Q11	0.967	15
Q19	0.843	9

Q5—question 5, Q6—question 6, Q9—question 9, Q10—question 10, Q11—question 11, and Q19—question 19.

**Table 2:** Sociodemographic characteristics of the study group.

	No. Respondents	Percent	Cumulative Percentage
Age			
20–30 years	22	17.2	17.2
31–40 years	36	28.1	45.3
41–50 years	40	31.3	76.6
51–60 years	26	19.7	96.9
>60 years	4	3.1	100.0
Profession			
Physician	51	39.8	39.8
Nurse	65	50.8	90.6
Psychologists and social workers	12	9.4	100.0
Gender			
Male	27	21.1	21.1
Female	101	78.9	100.0
Years of professional experience in patient care			
Under 5 years	39	30.5	30.5
5–10 years	29	22.7	53.1
11–20 years	33	25.8	78.9
over 20 years	27	21.1	100

**Table 3:** Knowledge of the concept of end-of-life therapeutic obstinacy.

Are You Familiar with the Concept of End-of-Life Therapeutic Obstinacy? (Q1)					
		Frequency	Percent	Valid Percent	Cumulative Percent
Valid	YES	119	93	93	93
	NO	9	7	7	100
	Total	128	100	100	

Q1—question 1.

**Table 4:** Frequency of dealing with therapeutic obstinancy in practice (Q2) according to profession (D2).

			Physician	Nurse	Psychologists and Social Workers	Total
Q2	Never	Count	1	0	0	1
		% within D2	2.0%	0.0%	0.0%	0.8%
	Very rare	Count	4	10	3	17
		% within D2	8.0%	15.4%	25.0%	13.4%
	Rare	Count	9	10	6	25
		% within D2	18.0%	15.4%	50.0%	19.7%
	Often	Count	32	41	3	76
		% within D2	64.0%	63.1%	25.0%	59.8%
	Very often	Count	4	4	0	8
		% within D2	8.0%	6.2%	0.0%	6.3%
Total		Count	50	65	12	127
		% within D2	100.0%	100.0%	100.0%	100.0%

Q2—question 2; D2—profession.

**Table 5:** Interventions considered to be disproportionate at the end of life in relation to the benefits obtained.

Interventions	Never	Very Rare	Rare	Often	Very Often
	1	2	3	4	5
Cardiopulmonary resuscitation	20 (15.6%)	11 (8.6%)	24 (18.8%)	31 (24.2%)	42 (32.8%)
Mechanical ventilation	18 (14.1%)	16 (12.5%)	22 (17.2%)	35 (27.3%)	37 (28.9%)
Transfusion of blood derivatives	20 (15.6%)	25 (19.5%)	20 (15.6%)	30 (23.4%)	33 (25.8%)
Parenteral nutrition	21 (16.4%)	17 (13.3%)	36 (28.1%)	38 (29.7%)	16 (12.5%)
Parenteral hydration	18 (14.1%)	19 (14.8%)	37 (28.9%)	38 (29.7%)	16 (12.5%)
Antibiotic therapy	17 (13.3%)	21 (16.4%)	32 (25.0%)	34 (26.6%)	24 (18.8%)
Haemodialysis	18 (14.1%)	23 (18.0%)	30 (23.4%)	37 (28.9%)	20 (15.6%)
Complex diagnostic investigations (CT/MRI/PET-CT)	20 (15.6%)	21 (16.4%)	21 (16.4%)	24 (18.8%)	42 (32.8%)
Paracentesis	15 (11.7%)	36 (28.1%)	38 (29.7%)	29 (22.7%)	10 (7.8%)
Thoracocentesis	15 (11.7%)	30 (23.4%)	42 (32.8%)	30 (23.4%)	11 (8.6%)
Establishment of nasogastric tube (NG tube) for feeding	11 (8.6%)	29 (22.7%)	36 (28.1%)	27 (21.1%)	25 (19.5%)
Establishment of NG tube for hydration	15 (11.7%)	19 (14.8%)	39 (30.5%)	31 (24.2%)	24 (18.8%)
Using existing NG tube for hydration and nutrition	16 (12.5%)	20 (15.6%)	41 (32.0%)	34 (26.6%)	17 (13.3%)
Gastrostomy/jejunostomy	17 (13.3%)	22 (17.2%)	29 (22.9%)	30 (23.4%)	30 (23.4%)
Nutrition and hydration on previously established gastrostomy tube	19 (14.8%)	19 (14.8%)	43 (33.6%)	40 (31.3%)	7 (5.5%)

CT—computer tomography; MRI—magnetic resonance imaging; PET-CT—Positron emission tomography; NG—nasogastric.

**Table 6:** Solutions considered to avoid situations of therapeutic obstinacy.

	Professions	Never	Very Rare	Rare	Often	Very Often
		1	2	3	4	5
Better communication between patient and…	physician	-	8 (6.3%)	3 (2.3%)	55 (43.0%)	62 (48.4%)
	nurse	1 (0.8%)	8 (6.3%)	9 (7.0%)	55 (43.0%)	55 (43.0%)
	psychologist	1 (0.8%)	9 (7.0%)	9 (7.0%)	51 (39.8%)	58 (45.3%)
	priest	1 (0.8%)	6 (4.7%)	12 (9.4%)	55 (43.0%)	54 (42.2%)
	social worker	-	8 (6.3%)	21 (16.4%)	54 (42.2%)	45 (35.2%)
Better communication between the patient’s family and…	physician	-	7 (5.5%)	9 (7.0%)	46 (35.9%)	66 (51.6%)
	nurse	1 (0.8%)	10 (7.8%)	11 (8.6%)	54 (42.2%)	52 (40.6%)
	psychologist	1 (0.8%)	13 (10.2%)	8 (6.3%)	54 (42.2%)	52 (40.6%)
	priest	1 (0.8%)	11 (8.6%)	16 (12.5%)	49 (38.3%)	51 (39.2%)
	social worker	2 (1.6%)	13 (10.2%)	18 (14.1%)	51 (39.8%)	44 (34.4%)
Informed consent explained and assumed by the patient		3 (2.3%)	10 (7.8%)	23 (18.0%)	49 (38.3%)	43 (33.6%)
Informed consent explained and assumed by the family on the patient’s request		1 (0.8%)	12 (9.4%)	20 (15.6%)	50 (39.1%)	45 (35.2%)
Legislative regulation on when CPR is not recommended		1 (0.8%)	8 (6.3%)	29 (22.7%)	39 (30.5%)	51 (39.8%)
Medical procedures and guidelines for these situations		2 (1.6%)	10 (7.8%)	23 (18.0%)	33 (25.8%)	60 (46.9%)
Adequate training of medical team members		2 (1.6%)	7 (5.5%)	17 (13.3%)	41 (32.0%)	61 (47.7%)

CPR—cardiopulmonary resuscitation.

## Data Availability

Data are contained within the article and Supplementary Materials.

## Supplementary Materials

The following supporting information can be downloaded at https://www.mdpi.com/article/10.3390/healthcare12161593/s1: Annex S1 (the questionnaire used in the research) and Annex S2 (The significance of the association between questions and professions (D2) is available).
