# Supplementary material for: Therapeutic Obstinacy in End-of-Life Care—A Perspective of Healthcare Professionals from Romania
Source: Healthcare (Basel). 2024 Aug 10;12(16):1593. doi: 10.3390/healthcare12161593 (PMC11353386; doi:10.3390/healthcare12161593)
Supplement: Supplementary file 1 [file healthcare-12-01593-s001.zip › healthcare-3110348-supplementary.pdf]

Annex S1

QUESTIONNAIRE THERAPEUTIC OBSTINACY AT THE END OF LIFE IN PALLIATIVE CARE SERVICES

Therapeutic obstinacy at the end of life (with a prognosis of a maximum of 72 hours of life) is defined as the unjustified persistence in unnecessary and disproportionate treatments in relation to the objectives and results reasonably expected for the health and/or improvement of the patient's quality of life, and only have the effect of prolonging the process of death.

The questionnaire is addressed to health professionals who care for patients at the end of life and aims to collect the opinions of specialists regarding the therapeutic intervention in end-of-life care.

As a result of your experience of caring for patients at the end of life and the complexity of the situations you encounter in your daily practice, please be kind enough to answer the following questions:

Age.: between:

20-30 years	31-40 years	41-50 years	51-60 years	>60 years
1	2	3	4	5

The profession:

Doctor		1
Specialty:		
Nurse		2
Psychologist		3
Priest/spiritual assistant		4
Social worker		5

Gender:

M	F
1	2

Years of professional experience in patient care:

Under 5 years	5-10 years	11 -20 years	over 20 years
1	2	3	4

Do you consider yourself a religious person?(please choose one answer)

Not at all	To a small extent	Neither too much nor too little	Largely	To a very large extent	I do not answer
1	2	3	4	5	6

1. **Are you familiar with the concept of therapeutic obstinacy?(please choose one answer)**

YES	NOT
1	2

2. **How often do you consider that you encounter therapeutic obstinacy in practice?**

(please choose one answer)

Never	Rarely	Rare	Often	Very often
1	2	3	4	5

3. **You have had personal experiences (with relatives/friends) that can be classified as "therapeutic obstinacy at the end of life":(please choose one answer)**

Never	Rarely	Rare	Often	Very often
1	2	3	4	5

4. In your opinion, which of the medical acts mentioned below can be included in the concept of therapeutic obstinacy at the end of life (you can choose one or more answer options):

Cardiopulmonary resuscitation		1
"Hospitalization" of the patient's end of life		2
Oxygen therapy		3
Surgical intervention for intestinal occlusion		4
Chemotherapy		5
Radiotherapy		6
Antibiotics therapy		7
Escalation of analgesia		8
None of the above		9
Other - please specify …………………………………..		10

5. **What do you think would be the pressure factors from the family of pacient in therapeutic obstinacy situations? (please rate each answer)**

		Never	Rarely	Rare	Often	Very often
		1	2	3	4	5
Family pressure	From the countryside					
	From the urban environment					
	From the diaspora					
the gender of the patient	Male					
	Female					
Age of the patient	< 18 years					
	18 – 45 years					
	45 – 65 years					
	65 -75 years					
	> 75 years					
The education level of the relative	No education/elementary education					
	With secondary education					

	With higher education					
Family religiosity	non-religious					
	religious					
The nature of the condition the patient is suffering from	Oncological conditions					
	Non-oncological conditions					
The desire to see a loved one for the last time						
Other factors: Nominate						

6. **What do you think would be the pressure factors from the patient's side in therapeutic obstinacy situations?(please rate each answer)**

		Never	Rarely	Rare	Often	Very often
		1	2	3	4	5
Patient pressure	From the countryside					
	From the urban environment					
	From the diaspora					
The gender of the patient	Male					
	Female					
Age of the patient	< 18 years					
	18 – 45 years					
	45 – 65 years					
	65 -75 years					
	> 75 years					
Patient's level of education	No education/elementary education					
	With secondary education					
	With higher education					
Religiosity of the patient	Non-religious patients					
	Religious patients					
The nature of the condition the patient is suffering from	Oncological conditions					
	Non-oncological conditions					
Other factors:						

Nominate						

7. **How important do you think is the pressure exerted by important people in society in situations of therapeutic obstinacy? (eg: people with important positions, dependent on social status)(please choose one answer)**

Not at all	To a small extent	Neither too much nor too little	Largely	To a very large extent
1	2	3	4	5

8. How important do you think professional experience is in managing these stressors?

Not at all	To a small extent	Neither too much nor too little	Largely	To a very large extent
1	2	3	4	5

9. In the care of the patient in the last 72 hours of life, which interventions do you consider to be disproportionate in relation to the benefits obtained?
(please rate each answer).

INTERVENTIONS	Never	Rarely	Rare	Often	Very often
	1	2	3	4	5
Cardiopulmonary resuscitation					
Mechanical ventilation					
Transfusion of blood products					
Parenteral nutrition					
Parenteral hydration					
antibiotics					
Hemodialysis					
Complex diagnostic investigations (CT/MRI/PET-CT)					
Paracentesis					
Thoracentesis					
Establishment of a nasogastric tube (SNG) - for feeding					
Institution of SNG- for hydration					
Use of already existing SNG for hydration and nutrition					
Establishment of a gastrostomy/jejunostomy					
Nutrition and hydration on the previously established gastrostomy					

None of the above					
Other - please specify					

10. What are the causes of therapeutic obstinacy in the last 72 hours of life? (please rate each answer)

		Never	Rarely	Rare	Often	Very often
		1	2	3	4	5
No knoledge of the diagnosis of the underlying disease by	patient					
	family					
	Doctor/care staff					
No knoledge of terminal condition diagnosis by	patient					
	family					
	Doctor/care staff					
Non-acceptance of the diagnosis of terminal condition by	patient					
	family					
	Doctor/care staff					
Not knowing of the prognosis of the disease by	patient					
	family					
	Doctor/care staff					
Failure to accept the prognosis by	patient					
	family					
Short time since diagnosis						
The emotional storm in the context of finding out the diagnosis or prognosis						
Poor collaboration/communication between the doctor and	patient					
	family					
Poor collaboration/communication between the nurse	patient					
	family					
A reduced degree of religiosity of	patient					
	Family					
	doctor					

An increased degree of religiosity of	patient					
	Family					
	doctor					
Fear in the face of death of the	patient					
	Family					
	doctor					
The family's fear of the patient's death						
Failure to express the patient's end-of-life preferences						
Not knowing of the patient's end-of-life preferences by the	family					
	doctor					
Medical staff's fear of malpractice claims from	patient					
	the patient's family					

11. What do you think would be the solutions to avoid therapeutic obstinacy situations? (please rate each answer):

		Never	Rarely	Rare	Often	Very often
		1	2	3	4	5
Better communication between the patient and	doctor					
	the medical assistant					
	psychologist					
	priest					
	the social worker					
Better communication between the patient's family and	doctor					
	the medical assistant					
	psychologist					
	priest					
	the social worker					
Informed consent explained and assumed by the patient						
Informed consent explained and assumed by the family, at the patient's request						

Legislative regulation regarding cases in which cardiopulmonary resuscitation is not recommended						
Medical procedures and guidelines for these situations						
Adequate professional training of medical team members						
Other - please specify						

12. Do you agree with the continuation of a treatment disproportionate to the benefits in the last 72 hours of life?

Never	Rarely	Rare	Often	Very often
1	2	3	4	5

13. How necessary do you consider the psychological preparation of the patient to accept the prognosis?

not at all	to a small extent	neither too much nor too little	largely	to a very large extent
1	2	3	4	5

14. How necessary do you consider the spiritual preparation of the patient to accept death?

not at all	to a small extent	neither too much nor too little	largely	to a very large extent
1	2	3	4	5

15. How necessary do you consider the psychological preparation of the medical staff to accept the death of the patient?

not at all	to a small extent	neither too much nor too little	largely	to a very large extent
1	2	3	4	5

16. How necessary do you consider the spiritual preparation of the medical staff to accept the patient's death?

not at all	to a small extent	neither too much nor too little	largely	to a very large extent
1	2	3	4	5

17. How necessary do you consider the psychological preparation of the family to accept the death of the patient?

not at all	to a small extent	neither too much nor too little	largely	to a very large extent
1	2	3	4	5

18. How necessary do you consider the spiritual preparation of the family to accept the death of the patient?

not at all	to a small extent	neither too much nor too little	largely	to a very large extent
1	2	3	4	5

19. In your experience, what are the most common wishes of end-of-life patients? (please rate each alternative)

Wishes	never	rare	occasionally	often	always
	1	2	3	4	5
To be left at home, "to die in his bed"					
To give him pain medication					
May his family be by his side					
To be held by the hand					
Let a prayer be offered to him by those close to him					
To receive spiritual assistance (e.g. to receive confession and communion from a priest)					
Let a candle be lit for him					
To satisfy a whim					
Recreating a family environment for the patient in the hospital room					
Anything else - please specify					

20. If you wish to express certain comments:

....

Thank you for your kindness in answering our questions and we guarantee the confidentiality of what you say!

Note: CPR: Cardiopulmonary Resuscitation; CT: Computer Tomography; MRI: Magnetic Resonance Imaging; PET-CT: Positron Emission Tomography-Computed Tomography; NG: Nasogastric

Annex S2. Tabel. Significance of the association between questions and professions (D2)

Question	Calculated statistical test χ^2	Level of significance	Decision
**Frequency of situations of obstinacy (Q2)**	13.992	0.082	**There is association**
**1. Family pressure factors (Q5)**			
**Rural environment (Q5_1)**	10.324	0.243	No association
**urban environment (Q5_2)**	9.401	0.310	No association
**Diaspora environment (Q5_3)**	**18.455**	**0.018**	**There is association**
**Male patient gender (Q5_4)**	10.490	0.232	No association
**Female patient gender (Q5_5)**	10.061	0.261	No association
**Patient's age < 18 years (Q5_6)**	**15.739**	**0.046**	**There is association**
**Patient's age 18 – 45 years (Q5_7)**	**27.289**	**0.001**	**There is association**
**Patient's age 45-65 (Q5_8)**	11.915	0.155	No association
**Patient's age 65 -75 years (Q5_9)**	**27.215**	**0.001**	**There is association**
**Patient's age > 75 years (Q5_10)**	**13.593**	**0.093**	**There is association**
Relative's level of education *(Q5_11-Q5_13)*			
**No education/elementary education (Q5_11)**	12.281	0.139	No association
**With secondary education (Q5_12)**	10.980	0.203	No association
**With higher education (Q5_13)**	12.372	0.135	No association
**non-religious (Q5_14)**	**15.734**	**0.046**	**There is association**
**religious (Q5_15)**	10.273	0.246	No association
**Oncological conditions (Q5_16)**	9.387	0.311	No association
**Non- oncological conditions (Q5_17)**	9.387	0.311	No association
**Ethnicity (Q5_18)**	7.099	0.526	No association
**The desire to see a loved one for the last time (Q5_19)**	5.629	0.689	No association
**2. The role of experience in managing pressure factors (Q8)**	11.375	0.181	No association
**3. Interventions considered disproportionate to the benefits (Q9)**			
**Cardiopulmonary resuscitation (Q9_1)**	6.559	0.585	No association
**Mechanical ventilation (Q9_2)**	6.856	0.552	No association

**Transfusion of blood derivatives** (**Q9_3**)	9.730	0.285	No association
**Parenteral nutrition** (**Q9_4**)	12.705	0.122	No association
**Parenteral hydration** (**Q9_5**)	4.124	0.846	No association
**Antibiotic therapy** (**Q9_6**)	3.774	0.957	No association
**Haemodialysis** (**Q9_7**)	9.372	0.312	No association
**Complex diagnostic investigations (CT/MRI/PET-CT)** (**Q9_8**)	12.724	0.122	No association
**Paracentesis** (**Q9_9**)	4.105	0.848	No association
**Thoracocentesis** (**Q9_10**)	7.548	0.479	No association
**Establishment of nasogastric tube (SNG)- for feeding** (**Q9_11**)	6.181	0.627	No association
**Establishment of SNG- for hydration** (**Q9_12**)	**14.784**	**0.063**	**There is association**
**Use of existing SNG for hydration and feeding** (**Q9_13**)	12.503	0.130	No association
**Gastrostomy/ jejunostomy** (**Q9_14**)	11.229	0.189	No association
**Nutrition and hydration on previously established gastrostomy tube Q9_15**	6.289	0.615	No association
**4. causes of therapeutic obstinacy** (Q10)			
**Lack of knowledge of the diagnosis of the basic disease by the patient** (**Q10_1**)	7.822	0.451	No association
**Lack of knowledge of the diagnosis of the basic disease by the family** (**Q10_2**)	4.600	0.799	No association
**Physician's lack of knowledge of basic disease diagnosis** (**Q10_3**)	7.383	0.496	No association
**Patient's lack of knowledge of terminal diagnosis (Q10_4**)	4.345	0.825	No association
**Family 's lack of knowledge of terminal diagnosis (Q10_5**)	5.117	0.745	No association
**Physician's lack of knowledge of terminal diagnosis (Q10_6**)	7.806	0.453	No association
Patient**'s refusal to accept terminal diagnosis** (**Q10_7**)	1.111	0.997	No association
Family **not accepting the diagnosis of terminal illness (Q10_8**)	5.765	0.674	No association
Physician's **failure to accept terminal diagnosis** (**Q10_9**)	7.640	0.469	No association
Patient**'s lack of knowledge of disease prognosis** (**Q10_10**)	**13.420**	**0.098**	**There is association**

**Lack of knowledge of the prognosis of the disease by the** family (**Q10_11**)	6.841	0.554	No association
Physician's **lack of knowledge of disease prognosis** (**Q10_12**)	11.403	0.180	No association
**Patient's non-acceptance of prognosis (Q10_13**)	6.448	0.597	No association
**Non-acceptance of prognosis by family (Q10_14**)	10.440	0.236	No association
**Short time to diagnosis** (**Q10_15**)	12.294	0.139	No association
**Emotional storm in the context of finding out the diagnosis or prognosis** (**Q10_16**)	**12.824**	**0.086**	**There is association**
**Poor collaboration/communication between physician and patient (Q10_17**)	6.398	0.603	No association
**Poor collaboration/communication between physician and family (Q10_18**)	7.818	0.451	No association
**Poor collaboration/communication between nurse and patient (Q10_19**)	7.286	0.506	No association
**Poor collaboration/communication between nurse and family (Q10_20**)	8.402	0.395	No association
**A low degree of patient's religiosity (Q10_21**)	**14.697**	**0.065**	**There is association**
**A low degree of family's religiosity Q10_22**	**13.759**	**0.088**	**There is association**
**A low degree of the physician's religiosity** (**Q10_23**)	**16.755**	**0.033**	**There is association**
**A high degree of patient's religiosity (Q10_24**)	7.013	0.535	No association
**A high degree of family's religiosity (Q10_25**)	13.136	0.107	No association
**A high degree of the physician's (Q10_26**)	2.386	0.967	No association
**the patient's fear of death.** (**Q10_27**)	**24.340**	**0.002**	**There is association**
Family **fear of death** (**Q10_28**)	**12.959**	**0.022**	**There is association**
Physician's **fear of death** (**Q10_29**)	**15.855**	**0.045**	**There is association**

**Family fear of patient's death** (**Q10_30**)	11.450	0.177	No association
**Not expressing patient's end-of-life preferences (Q10_31**)	10.571	0.227	No association
**Lack of knowledge of patient's end-of-life preferences by family (Q10_32**)	6.637	0.576	No association
**Physician's lack of knowledge of patient's end-of-life preferences (Q10_33**)	5.110	0.746	No association
**Fear of medical staff regarding patient's malpractice complaints (Q10_34**)	**19.662**	**0.012**	**There is association**
**Fear of medical staff regarding malpractice complaints from patient's family (Q10_35**)	**24.713**	**0.002**	**There is association**
5. **Solutions to avoid situations of therapeutic obstinacy (Q11)**			
**Better communication between patient and physician (Q11_1)**	4.241	0.835	No association
**Better communication between patient and nurse (Q11_2)**	5.958	0.652	No association
**Better communication between patient and psychologist (Q11_3)**	3.155	0.924	No association
**Better communication between patient and priest (Q11_4)**	4.356	0.824	No association
**Better communication between patient and social worker (Q11_5)**	1.113	0.997	No association
**Better communication between the patient's family and Q11_6**	2.646	0.955	No association
**Better communication between the patient's family and Q11_7**	3.609	0.891	No association
**Better communication between the patient's family and Q11_8**	4.206	0.838	No association
**Better communication between the patient's family and Q11_9**	5.501	0.703	No association

**Better communication between the patient's family and Q11_10**	2.111	0.977	No association
**Informed consent explained and assumed by the patient Q11_11**	4.746	0.784	No association
**Informed consent explained and assumed by the family on the patient's request (Q11_12)**	3.774	0.877	No association
**Legislative regulation of when CPR is not recommended (Q11_13)**	**16.285**	**0.038**	**There is association**
**Medical procedures and guidelines for these situations (Q11_14)**	**19.251**	**0.014**	**There is association**
**Adequate training of medical team members (Q11_15)**	9.037	0.339	No association
**6. Agreement with continuing treatment disproportionate to the benefits in the last 72 hours of life (Q12)**	5.118	0.745	No association

*Note : Statistical analysis was conducted using SPSS version 18.0. The statistical test performed is the Chi-square test (χ^2), used to determine the association between categorical variables. p-value (level of significance) less than 0.10 indicates that the association is statistically significant; p-value (level of significance) greater than 0.10 indicates that the association is not statistically significant; - Q: Question (for example, Q2 refers to the second question in the questionnaire); CPR: Cardiopulmonary Resuscitation; CT: Computer Tomography; MRI: Magnetic Resonance Imaging; PET-CT: Positron Emission Tomography-Computed Tomography; NG: Nasogastric
